# Dynamic Interplay between Cockayne Syndrome Protein B and Poly(ADP-Ribose) Polymerase 1 during Oxidative DNA Damage Repair

**DOI:** 10.3390/biomedicines10020361

**Published:** 2022-02-02

**Authors:** Robert J. Lake, Rabeya Bilkis, Hua-Ying Fan

**Affiliations:** Division of Molecular Medicine, Department of Internal Medicine, University of New Mexico Comprehensive Cancer Center, Albuquerque, NM 87131, USA; rjlake@salud.unm.edu (R.J.L.); rabilkis@salud.unm.edu (R.B.)

**Keywords:** Cockayne syndrome group B protein (CSB), poly(ADP-ribose) polymerase 1 (PARP1), oxidative stress, PARylation, Cockayne syndrome, ATP-dependent chromatin remodeler, DNA repair, oxidative stress-induced chromatin association

## Abstract

Oxidative stress contributes to numerous diseases, including cancer. CSB is an ATP-dependent chromatin remodeler critical for oxidative stress relief. PARP1 is the major sensor for DNA breaks and fundamental for efficient single-strand break repair. DNA breaks activate PARP1, leading to the synthesis of poly(ADP-ribose) (PAR) on itself and neighboring proteins, which is crucial for the recruitment of DNA repair machinery. CSB and PARP1 interact; however, how CSB mechanistically participates in oxidative DNA damage repair mediated by PARP1 remains unclear. Using chromatin immunoprecipitation followed by quantitative PCR, we found that CSB and PARP1 facilitate each other’s chromatin association during the onset of oxidative stress, and that CSB facilitates PARP1 removal when the level of chromatin-bound CSB increases. Furthermore, by monitoring chromatin PAR levels using Western blot analysis, we found that CSB sustains the DNA damage signal initiated by PARP1, and may prevent PARP1 overactivation by facilitating DNA repair. By assaying cell viability in response to oxidative stress, we further demonstrate that PARP1 regulation by CSB is a major CSB function in oxidatively-stressed cells. Together, our study uncovers a dynamic interplay between CSB and PARP1 that is critical for oxidative stress relief.

## 1. Introduction

The average human cell is estimated to receive about 20,000 DNA-damaging events daily through reactive oxygen species (ROS) generated from normal metabolic processes [1,2]. If not repaired efficiently, DNA damage will lead to genome instability, which can result in cell death or disease, such as cancer. The hydroxyl radical is the major cause of ROS-induced DNA damage; it attacks the sugar of the phosphodiester backbone as well as DNA bases. These two types of DNA lesions are repaired by single-strand break repair (SSBR) and base-excision repair (BER), respectively. 

Poly(ADP-ribose) polymerases (PARPs) comprise a large protein family that catalyzes the transfer of ADP-ribose from NAD^+^ to protein substrates, a process termed PARylation [3]. The human PARP family contains 17 members; among them PARP1 is the most abundant and accounts for ~90% of the PARylation activity in a cell. PARP1 is the major sensor for DNA breaks, and plays a critical role in SSBR. Initiation of SSBR is achieved by the rapid localization of PARP1 to SSBs. BER starts with base excision by a DNA glycosylase, followed by a common pathway usually involving an AP-endonuclease that generates DNA nicks. The importance of PARP1 in BER is still controversial, as it is believed that once the nick is generated, the repair enzymes work collectively and efficiently, so PARP1 is not needed as a responder [4,5,6].

Once bound to DNA breaks, PARP1’s enzymatic activity is stimulated, leading to PARylation of itself as well as other proteins, such as histones [7]. A large body of evidence indicates that PARylation contributes to the recruitment of DNA repair machinery. Auto PARylation of PARP1 has also been suggested to promote PARP1 dissociation from DNA after lesion detection to permit DNA repair [6]. The crucial role of PARP1 in DNA repair renders PARP1 a valuable target in cancer therapy, as PARP inhibitors increase the sensitivity of tumor cells to DNA damaging agents, especially those cells that are defective in homologous recombination repair. To date, PARP1 inhibitors target the catalytic domain; however, other mechanisms of PARP1 pathway inhibition might offer promise for therapeutic intervention in cancer as well as non-oncogenic diseases [8].

Cockayne syndrome complementation group B protein (CSB) is a member of the Swi2/Snf2 ATP-dependent chromatin remodeler family [9]. These proteins are fundamental to all nuclear processes involving DNA, as they regulate DNA access in chromatin [10]. They use ATP as energy to reposition histone octamers on DNA as well as dissociate non-histone proteins from chromatin [11,12,13]. 

Mutations in CSB account for ~80% of Cockayne syndrome cases, a premature aging syndrome in which patients suffer from numerous developmental and neurological abnormalities as well as extreme sun sensitivity. CSB is involved in both gene regulation and DNA repair [9,14,15]. In general, CSB interacts with chromatin dynamically; however, upon genotoxic stress, such as exposure to UV irradiation or oxidizing agents, a stable CSB–chromatin association is induced [16,17]. CSB is essential for transcription-coupled nucleotide excision repair (TC-NER), a process that removes transcription-stalling DNA lesions, such as those created by UV irradiation [18,19,20]. Previously, we found that ATP hydrolysis by CSB is necessary for the targeting of CSB to DNA lesion-stalled transcription, which is necessary to initiate TC-NER [16]. CSB is required for the recruitment of DNA repair machinery, independent of its chromatin remodeling activity. Instead, its remodeling activity has been hypothesized to create a chromatin environment for efficient DNA repair or transcription resumption after repair [21]. In strong contrast, we found that stable CSB–chromatin association induced by oxidative stress does not require ATP hydrolysis by CSB; however, the PARP1 protein is required for efficient CSB recruitment, underscoring the importance of PARP1 in regulating CSB targeting in oxidatively-stressed cells [22]. 

CSB is important for the repair of oxidative stress-induced DNA lesions, as oxidative DNA lesions are increased in cells derived from Cockayne syndrome patients, and CSB has been found to functionally interact with 8-oxoguanine DNA glycosylase (OGG1) as well as apurinic/apyrimidinic endonuclease 1 (APE1), two enzymes critical for BER [23,24,25,26,27]. Moreover, CSB has been found to facilitate the recruitment of X-ray repair cross complementing 1 (XRCC1) to oxidative DNA damage [28]. 

Our previous work indicated that CSB also functions in single-strand break repair by collaborating with PARP1 [22]. Specifically, PARP1 increased the association kinetics of CSB to oxidatively damaged chromatin independently of PARP1’s enzymatic activity [22]. Thorslund et al. (2005) demonstrated that CSB interacts with PARP1 in vitro, that these two proteins can colocalize in cells, and that CSB is PARylated by PARP1 in oxidatively-stressed cells [29]. Results from in vitro assays have led to the suggestions that CSB may regulate PARP1 activity by facilitating its dissociation from DNA [30]. 

In our earlier study, we identified oxidative stress-induced CSB occupancy sites by anti-CSB chromatin immunoprecipitation followed by deep sequencing (CSB ChIP-seq) [17]. In our present study, we used the top four CSB binding sites (i.e., chrX-1, chrX-2, chr17-1 and chr19-2) as experimental paradigms and uncovered an intimate crosstalk between CSB and PARP1 at the levels of both chromatin association and activity regulation in oxidatively-stressed cells. By extending our analysis, we show that the crosstalk between CSB and PARP1 occurs at a genome-wide level. Significantly, our study reveals that this crosstalk is important to cell viability in response to oxidative stress. 

## 2. Materials and Methods

### 2.1. Cell Culture and Menadione Treatment

CS1AN and CSB^WT^ cells were maintained in DMEM-F12 medium supplemented with 10% FBS [31]. 293T cells were maintained in DMEM supplemented with 10% FBS. All cells were maintained at 37 °C, 5% CO_2_. Human hTERT RPE-1 cell lines were maintained according to Hanzlikove et al. (2017) [32]. Oxidative stress was induced by treating cells with menadione (#102259, MP Biomedicals, Solon, OH, USA) in fresh medium and incubated at 37 °C for the time points indicated.

### 2.2. Protein Fractionation and Western Blotting

Approximately 1.5 million cells were seeded onto each 60 mm dish. The following day, 100 μM menadione was used to treat cells for the indicated durations. Cells were then lysed, and proteins were fractionated as described previously [16,22]. Briefly, cells were rinsed with PBS and lysed in 200 μL buffer B (20 mM HEPES (pH 8.0), 0.5 mM MgCl_2_, 150 mM NaCl, 10% glycerol, 0.5% Triton X-100, 1 mM DTT). Cell lysate was collected on ice using cell lifters, and lysates were centrifuged at ~21,000 *g* for 20 min at 4 °C. The soluble fraction (S) was generated by mixing 150 μL of the supernatant with 50 μL 4× SDS sample buffer. The chromatin-enriched fraction was generated by adding 200 μL of 1× SDS sample buffer to the pellet, which was subsequently sonicated for 60 s at 25% amplitude with a Branson 101-135-126 sonifier. The resulting chromatin fraction was 1.33 times more concentrated than the soluble fraction (S). 

For protein quantification, 200 μL of 1× SDS sample buffer without DTT and bromophenol blue was added to the pellet, which was sonicated for 60 s at 25% amplitude with a Branson 101-135-126 sonifier and quantified using a BCA protein assay kit (Thermo Fisher, Waltham, MA, USA) according to the manufacturer’s instructions. Approximately 20 μg of protein was loaded per lane on a 4–12% Bis-Tris SurePAGE gel (GenScript, NJ, USA). Western blots were analyzed using antibodies described below, developed using SuperSignal West Pico or Dura chemiluminescent substrates (Thermo Fisher), and processed with a Konica Processor SRX-101A. Films were scanned and quantified using ImageQuantTL (V10.0.261, Cytiva, Marlborough, MA, USA).

### 2.3. shRNA Knockdown

Mission shRNA targeting CSB (TRCN0000016775) and a nontargeting shRNA (SHC002) were purchased from Sigma-Aldrich (St. Louis, MO, USA). Lentivirus was produced by co-transfecting 293T cells (~90% confluent) with the indicated shRNA and third-generation packaging plasmids (pMGLg-RRE, pRSV-REV and pMD2.G/VSV). The culture medium was changed 24 h post-transfection, and virus-containing medium was collected after another 24 h incubation. Target RPE cells were ~20% confluent at the time of infection. The medium was changed 24 h after infection, and cells were harvested at 72 h post-infection for Western blot analysis or seeded for cell survival assays.

### 2.4. Menadione Sensitivity Assays

Approximately 2 × 10^−5^ cells were seeded onto each 60 mm plate. The following day, cells were treated with menadione at the indicated concentrations for 1 h, and the cells were incubated for 24 h in fresh medium. Trypan-blue was used to score live versus dead cells [22]. The numbers of clear (live) and blue (dead) cells were counted using a hemocytometer. 

### 2.5. ChIP-qPCR Analyses

Chromatin immunoprecipitation (ChIP) was carried out as previously described [31]. Briefly, approximately 4 million cells were fixed with 1% formaldehyde for 10 min and sonicated on ice at 40% amplitude (30 s on, 90 s off for 24 min) using a Branson 101-135-126 sonifier. ChIP was performed using a monoclonal anti-CSB antibody (1B1) (1:25) and 5 μL blocked protein-G agarose beads (VWR, Radnor, PA, USA). Samples were reverse crosslinked for 16 h at 65 °C. ChIPed DNA was purified and analyzed by real-time PCR, using PerfeCTa SYBR Green FastMix, Low ROX (QuantaBio, Beverly, MA, USA) in a 384-well format with a QuantStudio 5 real-time PCR system (Applied Biosystems, Waltham, MA, USA). Real-time PCR data were analyzed using the ΔΔCt method [33]. Primers used are as described previously [17]. For ADP-ribose chromatin affinity precipitation (ADPr-ChAP) cells were processed as described above for ChIP, except that the pan-PAR binding reagent (MABE1016, EMD Millipore, Burlington, MA, USA) was used instead of antibody, at a dilution of 1:100 [34].

### 2.6. Antibodies

Antibodies used for Western blot analysis were rabbit polyclonal anti-CSB (N-terminus or C-terminus) (1:2000) (provided by Dr. Weiner, University of Washington), XRCC1 (NB120-1838, 1:100) (Novus Biologicals, Centennial, CO, USA), lamin B1 (#13435, 1:300) (Cell Signaling Technology, Danvers, MA, USA), rabbit polyclonal anti-histone H3 total (#9715, 1:2000) (Cell Signaling Technology, Danvers, MA, USA), mouse monoclonal anti-GAPDH (1:10,000) (Millipore, MAB374), HRP-conjugated goat anti-rabbit IgG (31460, 1:10,000) (Pierce, Rockford, IL, USA), and HRP-conjugated goat anti-mouse (IgG + IgM) (115-035-044, 1:10,000) (Jackson Laboratory, Bar Harbor, ME, USA). ChIP was performed using a monoclonal mouse anti-CSB antibody (1B1), which has been mapped to the N-terminal 507 amino acids of CSB [31], as well as a rabbit polyclonal antibody against PARP1-C (Active motif, #39561, Carlsbd, CA, USA). A pan ADP-ribose-binding reagent (MABE1016, EMD Millipore, Burlington, MA, USA) was used to detect PAR in both Western blot analyses and ADPr-ChAP assays [34].

## 3. Results

### 3.1. PARP1 Is Essential for CSB Recruitment to the Top Four Menadione-Induced CSB Binding Sites

**Figure 1:**
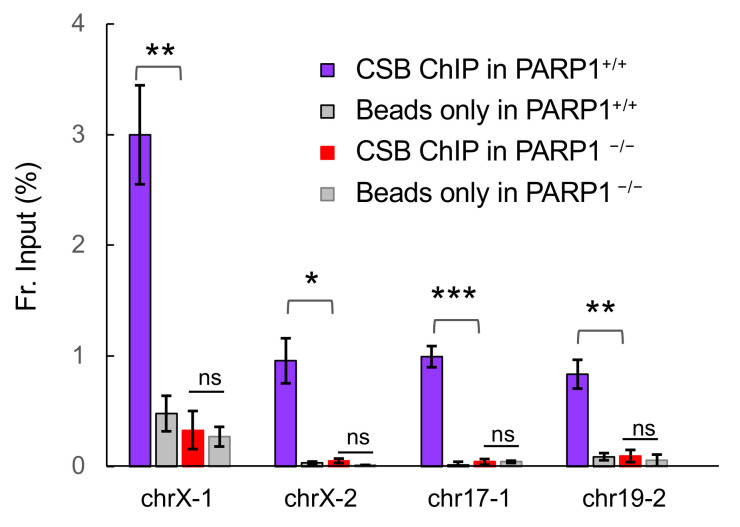
PARP1 is essential for the recruitment of CSB to the top four oxidative stress-induced CSB occupancy sites. CSB ChIP-qPCR was carried out in PARP1^+/+^ and PARP1^−/−^ RPE cells after a 1 h, 100 μM menadione treatment. Shown is CSB enrichment at the top four menadione-induced CSB binding sites, using primers within the peak binding regions [17]. Data are presented as means +/− SEM (*n* = 3 biological replicates). Paired *t*-tests were used to compare CSB enrichment between cells with and without PARP1; *: *p* < 0.05, **: *p* < 0.01, ***: *p* < 0.001.

ChrX-1, chrX-2, chr17-1 and chr19-2 are the top four CSB binding sites induced by menadione (Figure 1) [31]. We found that these four regions are uniquely different from CSB binding sites used for transcriptional regulation, which we also identified [31]. Specifically, CSB enrichment at these sites is much higher (~300 vs. ~30 reads per million (rpm)) and the width of CSB occupancy is larger (0.9–1.5 vs. ~0.2 Kb) than sites for transcription regulation. We found that both coding and non-coding DNA in and around each site displays very low transcriptional activity in the presence or absence of oxidative stress (not shown). We previously found that PARP1 positively regulates the recruitment of CSB to these sites, which is to a large degree independent of PARP1 enzymatic activity [22]. To study further how critical PARP1 is in regulating menadione-induced CSB recruitment to these loci, we performed anti-CSB ChIP-qPCR in the near-diploid retinal pigment epithelial (RPE) cell line (PARP1^+/+^) and the isogenic RPE cell line in which both PARP1 alleles were deleted by CRISPR (PARP1^−/−^) [32]. As shown in Figure 1, CSB is recruited to these sites in PARP1^+/+^ RPE cells treated with menadione (purple), similar to that observed in CSB^WT^ cells [17]. Strikingly, we found no significant CSB recruitment in PARP1^−/−^ cells (red). These observations extend our previous finding in fibroblasts to epithelial cells and further demonstrate that PARP1 is absolutely required for the recruitment of CSB to specific genomic loci in menadione-treated RPE cells. 

### 3.2. CSB Regulates the Association of PARP1 with Chromatin upon Oxidative Stress

**Figure 2:**
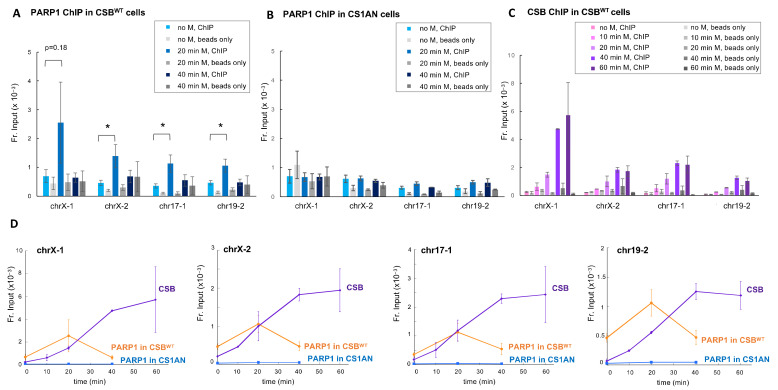
Transient PARP1 recruitment to menadione-induced CSB binding sites is CSB dependent. (**A**) PARP1 ChIP-qPCR in CSB^WT^ cells and (**B**) CS1AN cells at different time points during 100 µM menadione treatment. Paired *t*-tests were used to compare PARP1 enrichment at 20 min of menadione treatment to no treatment; *: *p*< 0.05. No significant PARP1 enrichment over “beads-only” control at 40 min of treatment in CSB^WT^ cells. No significant PARP1 enrichment over “beads-only” control at all time points in CS1AN cells. (**C**) CSB ChIP-qPCR in CSB^WT^ cells. (**D**) Graphical overlay of PARP1 and CSB occupancy at the four loci analyzed in CSB^WT^ and CS1AN cells. Shown are means +/− SEM (*n* = 3 biological replicates).

We next determined if menadione treatment induces the recruitment of PARP1 to specific CSB binding sites using anti-PARP1 ChIP-qPCR. In these experiments, we compared the CSB functional null cell line, CS1AN, to CSB^WT^ cells, which are CS1AN cells reconstituted with CSB. As shown in Figure 2A, we found an increase in PARP1 occupancy at these sites after a 20 min menadione treatment in CSB^WT^ cells, but no significant PARP1 occupancy at 40 min of treatment. Strikingly, we did not detect any significant PARP1 occupancy at these regions in CS1AN cells (Figure 2B), demonstrating that CSB positively regulates the interaction of PAPR1 with chromatin at these CSB binding sites within 20 min of treatment. To gain more insight into the interplay between CSB and PARP1 chromatin association, we examined CSB occupancy at these regions in a time-dependent manner. As shown in Figure 2C, the occupancy of CSB at these regions gradually increased and reached a steady-state level around 40 min. Figure 2D is a graphical overlay of CSB and PARP1 occupancy at each locus. These data reveal that CSB positively regulates the interaction of PARP1 with these genomic regions during the onset of oxidative stress when the local CSB concentration on chromatin is relatively low, and suggests that when CSB becomes more abundant, CSB promotes PARP1 dissociation from chromatin. 

### 3.3. CSB Regulates PAR Levels on Chromatin in Oxidatively-Stressed Cells

**Figure 3:**
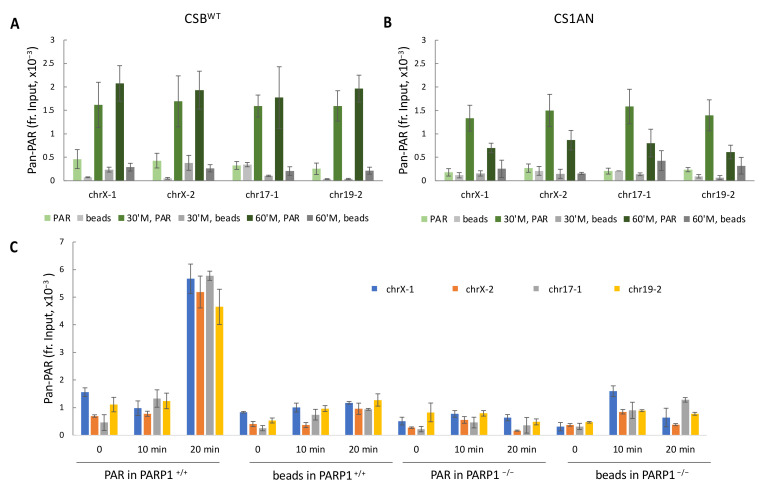
CSB regulates PAR levels at the top four CSB-binding sites induced by oxidative stress. (**A**–**C**) PAR levels at the four CSB binding sites during menadione treatment. A pan-PAR binding reagent was used to isolate PAR-enriched chromatin from formaldehyde crosslinked cells at the specified time points. The recovered chromatin was reverse-crosslinked and subjected to qPCR. (**A**) CSB^WT^ cells. (**B**) CS1AN cells. (**C**) Validation of the pan-PAR binding reagent using PARP1^−/−^ and PARP1^+/+^ RPE cells. PAR enrichment at these four regions was only significantly detected in PARP1^+/+^ cells after 20 min of menadione treatment. (UT = untreated).

We next monitored the signature of PARP1 activity at these regions by ADP-ribose chromatin affinity precipitation (ADPr-ChAP), using a pan-PAR binding reagent, which detects mono-, oligo- and poly-ADP-ribose [34]. In CSB^WT^ cells, an increased PAR level was observed at 30 min after the onset of menadione treatment, and this increase was sustained at 60 min of treatment (Figure 3A). Similarly, in CS1AN cells, we also detected an increased PAR level at these regions after 30 min of treatment; however, the PAR signals at these regions dropped at 60 min of treatment (Figure 3B). We verified the specificity of the pan-PAR binding reagent by repeating this experiment in PARP1^+/+^ and PARP1^−/−^ RPE cells. Within the first 10 min of menadione treatment, we did not detect a significant PAR signal at these regions over the “beads-only” controls in PARP1^+/+^ cells. However, a dramatic increase in PAR signal was detected after 20 min of menadione treatment in PARP1^+/+^ cells, while no significant PAR signal over a “beads-only” control was detected in PARP1^−/−^ cells (Figure 3C), indicating the specificity of the PAR binding reagent.

Our results reveal that PARP1 can interact with oxidized chromatin in the presence or absence of CSB, as we detected similar PAR levels at the four CSB binding sites in both CSB^WT^ and CS1AN cells after a 30 min menadione treatment (Figure 3). However, since we only detected PARP1–chromatin association in CSB^WT^ cells but not in CS1AN cells (Figure 2), these data indicate that the interaction of PARP1 with oxidized chromatin is highly dynamic in the absence of CSB. CSB may promote the stabilization of PARP1 with oxidized chromatin at these loci, which may account for the slower decay of chromatin PAR levels that we observed.

**Figure 4:**
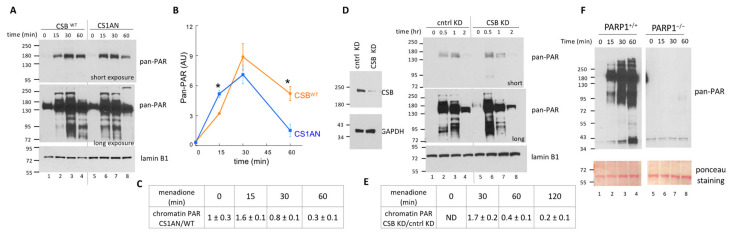
CSB regulates PAR levels on oxidatively damaged chromatin. (**A**) Chromatin-enriched fractions were isolated from CSB^WT^ and CS1AN cells at the indicated time points during a 100 µM menadione treatment. Equal amounts of chromatin-associated proteins at each time point were resolved by SDS-PAGE and PARylated proteins were identified with the pan-PAR binding reagent. Lamin B1 was used as a loading control. (**B**) Quantification of data from Western blots obtained from three biological replicates. Paired *t*-tests were used to compare PAR levels in CSB^WT^ vs. CS1AN cells; *: *p*< 0.05. (**C**) Ratios of PAR levels in CS1AN vs. CSB^WT^. Shown are means +/− SEM (*n* = 3 biological replicates). (**D**,**E**) Same as A and C, except that PARP1^+/+^ RPE cells expressing CSB-targeting shRNA were compared to cells expressing a control shRNA (*n* = 2 biological replicates). The extent of CSB knockdown is shown in the left panel. (**F**) Same as D, but PARP1^+/+^ and PARP1^−/−^ cells were used to demonstrate specificity of the PAR-binding reagent. Ponceau staining was used as loading control.

To examine the generality of PARP1 regulation by CSB, we monitored PAR levels on total chromatin using subcellular fractionation followed by Western blot analysis (Figure 4). Chromatin was isolated from menadione-treated CS1AN and CSB^WT^ cells after different durations of menadione treatment. Protein concentrations were measured, and equal amounts of protein were loaded onto an SDS-polyacrylamide gel. In both CS1AN and CSB^WT^ cells, we detected a significant increase in chromatin-associated PAR after 15 min of menadione treatment that continued to increase up 30 min of treatment. At 60 min of treatment, the PAR levels in both cell lines dropped; however, the chromatin PAR level in CSB^WT^ remained higher at 60 min as compared to CS1AN cells, which dropped to near basal level (Figure 4A). Averaged from three biological replicates, there was ~60% more PAR on CS1AN chromatin as compared to CSB^WT^ after 15 min of treatment. However, there was a ~70% reduction in chromatin PAR levels in CS1AN cells as compared to CSB^WT^ cells at 60 min treatment (Figure 4B,C). Similar results were obtained with the RPE PARP1^+/+^ cell line using shRNA targeting CSB: ~70% more chromatin-associated PAR was observed in CSB KD cells as compared to control KD cells after 30 min of menadione treatment, while there was an ~80% decrease in PAR level in CSB KD after 2 h of menadione treatment (Figure 4D,E). We again tested the specificity of the pan-PAR binding reagent by Western blot analysis. As shown in Figure 4F, we detected increasing levels of pan-PAR signal during the first hour of menadione treatment in RPE PARP1^+/+^ cells, while the pan-PAR signal was substantially diminished in PARP1^−/−^ cells, indicating that the vast majority of the PAR signal detected in Figure 4A,D is PARP1 dependent. Together, these results support the hypothesis that CSB regulates PARP1 activity during oxidative stress. Specifically, (1) CSB prevents PARP1 overactivation at the onset of oxidative stress, but (2) CSB is required to sustain PAR levels on chromatin at later times.

### 3.4. CSB Facilitates the Recruitment of XRCC1 to Oxidatively Damaged Chromatin

**Figure 5:**
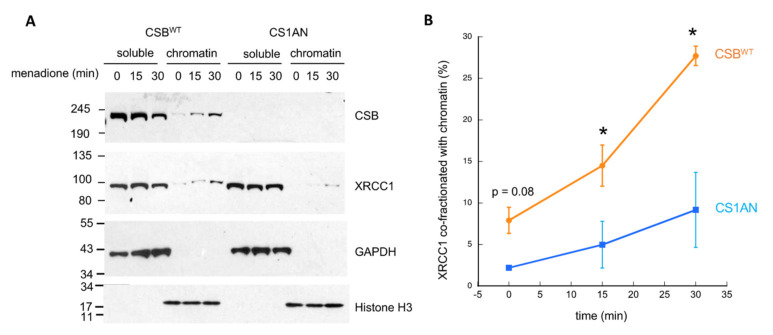
CSB increases the kinetics of XRCC1 association with oxidatively damaged chromatin. (**A**) Soluble and chromatin-enriched fractions from CSB^WT^ and CS1AN cells, treated with 100 µM menadione for the indicated time points, were resolved by SDS-PAGE and subjected to Western blot analysis. (**B**) Quantified Western blot signals were plotted as percent XRCC1 co-fractionating with chromatin. Shown are means +/− SEM (*n* = 3 biological replicates). *: *p*< 0.05.

PARylation is involved in the recruitment of DNA repair proteins. If CSB is involved in sustaining PARylation of oxidatively damaged chromatin, we expect to see facilitation of DNA repair machinery recruitment by CSB. Accordingly, we examined how CSB impacts the association kinetics of XRCC1 with oxidatively damaged chromatin (Figure 5). CSB^WT^ and CS1AN cells were treated with menadione for different lengths of time, subjected to subcellular fractionation, and then chromatin-associated proteins were analyzed by Western blot. In this assay, CSB functioned as the positive control for recruitment to oxidatively damaged chromatin, while GAPDH and histone H3 were used as loading controls for soluble and chromatin-enriched fractions, respectively. As shown in Figure 5A, XRCC1 displayed increased chromatin association as a function of time. Strikingly, the kinetics of this association was significantly decreased in CS1AN cells. Figure 5B shows the quantification of Western blot data from three biological replicates. These observations reveal that CSB enhances the recruitment of XRCC1 to chromatin upon oxidative stress. However, since chromatin PAR levels are similar at 30 min of menadione treatment in cells with or without CSB (Figure 4), these results indicate that the enhanced recruitment of XRCC1 to chromatin mediated by CSB does not rely on chromatin PAR levels. Nonetheless, this result does not exclude the possibility that chromatin PAR levels contribute to the stabilization of XRCC1 on chromatin once recruited (see Discussion).

### 3.5. Regulation of PARP1 Activity Is a Major CSB Function in Oxidatively-Stressed Cells

**Figure 6:**
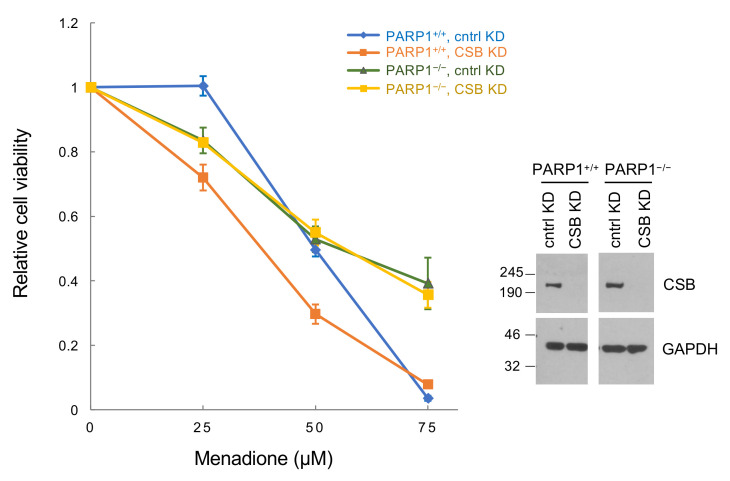
Menadione sensitivity assays. PARP1^+/+^ and PARP1^−/−^ RPE cells expressing shRNA targeting CSB or a control shRNA were treated with menadione at the indicated concentrations for 1 h. Cell viability was determined by trypan blue exclusion 24 h after treatment. The extent of CSB knockdown is shown to the right. Shown are means +/− SEM from three biological replicates.

To test how critical the interplay between CSB and PARP1 is in oxidatively-stressed cells, we performed cell-viability assays in RPE cells without PARP1, with decreased CSB levels (shRNA) or both. As shown in Figure 6, PARP1^+/+^ cells expressing shRNA targeting CSB were significantly more sensitive to menadione as compared to cells expressing control shRNA. Remarkably, knocking down CSB in PARP1^−/−^ cells did not make these cells more sensitive to menadione. These results reveal that, upon oxidative stress, CSB is more critical in cells with PARP1 than without PARP1, highlighting the significance of PARP1 regulation by CSB in oxidatively-stressed cells.

## 4. Discussion

Here we describe an intimate crosstalk between CSB and PARP1 in oxidatively-stressed cells. PARP1 is essential for the recruitment of CSB to the top four oxidative stress-induced CSB-binding sites (chrX-1, chrX-2, chr17-1 and chr19-2) (Figure 1). Reciprocally, CSB facilitates PARP1 association with these regions (Figure 2) when local CSB concentrations on chromatin are low. Of great interest, CSB promotes the dissociation of PARP1 from these regions when local CSB concentrations become higher. By measuring PARylation levels on oxidatively damaged chromatin, we demonstrate that CSB sustains chromatin PARylation initiated by PARP1 (Figure 3 and Figure 4) and prevents the overactivation of PARP1 during the onset of oxidative stress (Figure 4). Consistent with these results, we found that CSB increases the kinetics of XRCC1’s association with oxidized chromatin (Figure 5). Remarkably, our results from cell-viability assays underscore the significance of CSB function in PAPR1 activity regulation during oxidative stress, as decreasing CSB levels in PARP1^−/−^ cells did not make these cells more sensitive to oxidative stress, which is in stark contrast to PARP1^+/+^ cells that became more sensitive (Figure 6).

**Figure 7:**
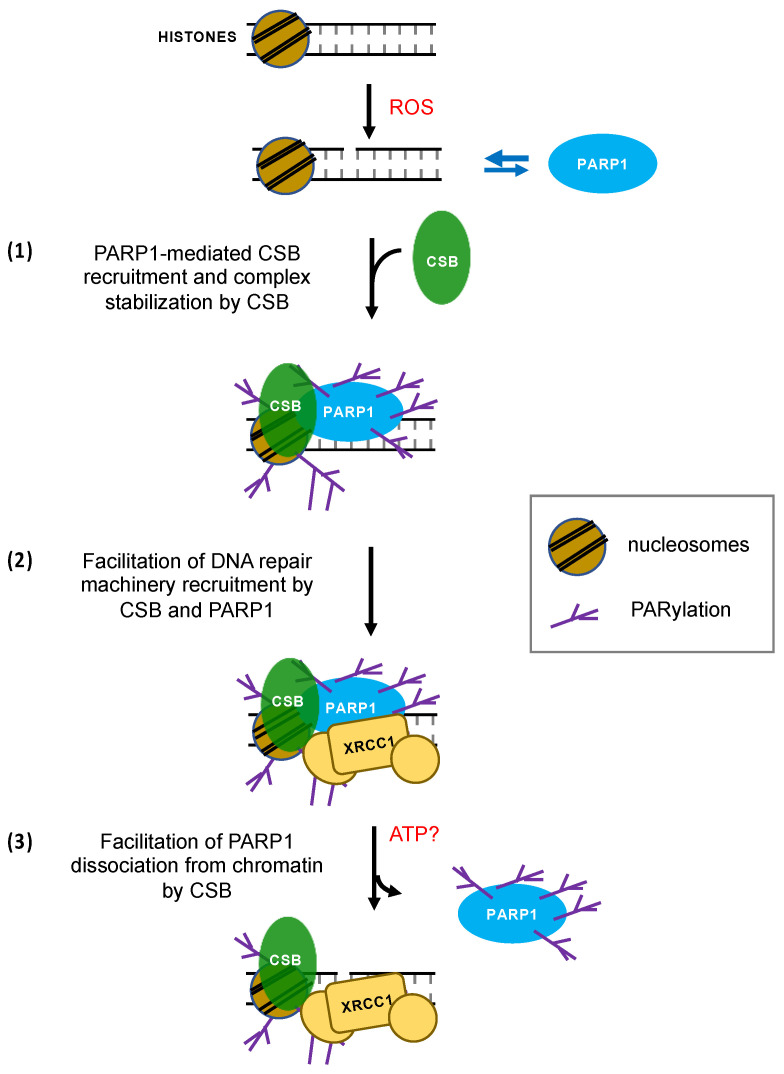
Model for the co-regulations of PARP1 and CSB activity during oxidative stress. In the absence of oxidative stress, both CSB and PARP1 interact with chromatin dynamically. However, in the presence ROS, SSBs are generated, which lead to PARP1 recruitment and activation. CSB interacts with both PARP1 and chromatin, and, together, these multivalent interactions increase the affinity of PARP1 and CSB for damaged chromatin step (1). Once activated by DNA breaks, PARP1 may PARylate CSB, in addition to itself and neighboring proteins, such as histones. The concerted action of PARP1 and CSB facilitates the recruitment of DNA repair proteins, such as XRCC1 step (2). As oxidative stress ensues, PARP1 dissociates from chromatin while CSB remains chromatin-bound step (3), sustaining the PARylation signal and facilitating efficient DNA repair. CSB may help displace PARP1 from chromatin to further enhance DNA repair and/or remodel nucleosomes to create a repair-conducive environment.

Results from our earlier study strongly suggest that CSB plays a role in SSBR, as PARP1 accelerates the association of CSB with oxidatively damaged chromatin, in contrast to OGG1 or APE1, the two major enzymes for BER, which had no effect on the kinetics of CSB–chromatin association [28]. Together, our results are consistent with a model whereby CSB and PARP1 coordinate their activities in DNA single-strand break repair (Figure 7) [22]. In the absence of DNA lesions, CSB and PARP1 interact with chromatin dynamically [16]. Upon oxidative stress, CSB and PARP1 positively regulate each other’s interaction with oxidatively damaged chromatin (Figure 7, step 1). Our observations indicate that at 20 min of menadione treatment, PARP1 is in the vicinity of oxidatively damaged chromatin, since we detected increased chromatin-associated PARylation, the signature of PARP1 enzymatic activity, in both CSB^WT^ and CS1AN cells. However, we only detected PARP1 protein occupancy in CSB^WT^ cells, indicating that PARP1 interacts with these regions in both CSB^WT^ and CS1AN cells, but CSB increases the residence time of PARP1 at these regions upon oxidative stress, possibly to enhance repair-protein recruitment. It is, nonetheless, possible that a stable association of PARP1 with chromatin may occur in CS1AN cells at very early times after the initiation of oxidative stress, and PARP1 is already released from chromatin by 20 min. A ChIP study with increased temporal resolution would address this possibility.

We hypothesize that PARP1-mediated recruitment of the DNA repair machinery occurs in a CSB-dependent manner (Figure 7, step 2). We showed that CSB enhances XRCC1–chromatin association at 30 min of menadione treatment (Figure 5), even though the chromatin-associated levels of PAR are similar in both CS1AN and CBS^WT^ cells at this time (Figure 4). Together, these observations indicate that CSB assists in the recruitment of XRCC1, independently of chromatin PARylation. Mechanistically, enhanced XRCC1 recruitment may be achieved through a direct CSB–XRCC1 association or indirectly through CSB’s chromatin remodeling activity, whereby a chromatin environment conducive for XRCC1 association is generated. Nonetheless, stabilization of XRCC1 association may ultimately be achieved through the interaction of XRCC1’s BRCT domain with PARylated proteins. Future studies will shed light on the mechanism by which CSB enhances XRCC1–chromatin association and how chromatin PAR levels sustained by CSB regulates oxidative DNA repair.

CSB subsequently facilitates the removal of PARP1 from DNA lesions to promote repair (Figure 7, step 3). Since we observed a slower decay of PAR levels in the presence of CSB (Figure 4), CSB may restrict the access of dePARylation enzymes, perhaps by steric interference. Alternatively, in the absence of CSB, PARP1 may be overactivated, and thus exhaust nuclear NAD^+^. It remains to be determined how the enzymatic activities of CSB are utilized in SSBR. We have shown that the ATPase activity is not required for CSB recruitment to oxidized chromatin [22]. However, we do not yet know if the ATP-dependent chromatin remodeling activity is required for the dissociation of PARP1 from oxidatively damaged DNA, for recruitment of the DNA repair machinery or providing an epigenetic landscape conducive for efficient DNA repair.

Why would PARP1^+/+^ cells be more sensitive to CSB levels than PARP1^−/−^ cells during oxidative stress (Figure 6)? One possible scenario is that CSB may regulate the enzymatic activity of PARP1, either directly through protein–protein interaction or indirectly by remodeling the chromatin environment (or both). This regulation may be necessary to prevent cell death resulting from NAD^+^ exhaustion, which will not occur as readily in PARP1^−/−^ cells. In the absence of PARP1, backup repair machinery that operates independently of CSB may take over; in this circumstance, CSB’s function in the regulation of PARP1 activity would no longer be required. Future experiments that measure NAD^+^ levels and DNA damage as well as employ in vitro reconstituted enzymatic assays will provide mechanistic and functional insights into the co-regulation of CSB and PARP1 activity in oxidatively-stressed cells.

Importantly, we observed similar changes in chromatin PAR levels in both fibroblast and epithelia cells, albeit at different times during the course of oxidative stress, which is consistent with the notion that different cell types have different tolerances to oxidative stress. Our observations indicate that CSB regulates PARP1-chromatin interactions and sustains chromatin PAR levels initiated by PARP1, thus expanding our understanding of how genome stability is maintained upon oxidative stress. Our results contrast with those from a study by Lee et al. (2019), in which they observed increased PAR levels one hour after menadione treatment in CS1AN cells as compared to CSB^WT^ cells [35] (Figure 4A–C). This discrepancy is most likely due to a difference in sample preparation. In the Lee et al. study, entire nuclear fractions were isolated from formaldehyde crosslinked cells and examined by Western blot; therefore, this study examined both chromatin-bound and -unbound PARylated proteins that reside in the nucleoplasm. In our study, we specifically examined chromatin fractions isolated under native conditions (non-crosslinked) (Figure 4). Our fractionation procedure, therefore, removed unbound proteins that reside in the nucleoplasm as well as the cytoplasm. Moreover, our ChIP analyses of the four CSB-occupied loci (Figure 2) examined chromatin fragments generated by sonication and, therefore, would again be enriched for chromatin-associated proteins. A difference in the PAR-binding and detection reagents used in these two studies may have also contributed to this discrepancy.

Previously, we hypothesized that chrX-1, chrX-2, chr17-1 and chr19-2 may represent sites where CSB regulates transcription in oxidatively-stressed cells; however, we have not observed any significant CSB-dependent changes in gene expression at these loci. Nonetheless, by using these loci as models, we found a dynamic interplay between CSB and PARP1 chromatin association and activity regulation. Importantly, we showed that the hypotheses derived from these model loci are applicable at the genome-wide scale (Figure 3 and Figure 4). Further analyses will reveal the extent to which these regions may be oxidative DNA lesion hotspots, and these loci will continue to serve as experimental paradigms to study the function of CSB in oxidative DNA damage repair.
